# Tissue microenvironment and benign prostatic hyperplasia

**DOI:** 10.18632/aging.102029

**Published:** 2019-06-13

**Authors:** Xing Wei, Li Xin

**Affiliations:** 1 Department of Urology University of Washington, Seattle, WA 98109, USA

**Keywords:** Wnt signaling, tissue microenvironment, stromal-epithelial interaction, benign prostatic hyperplasia

Benign prostatic hyperplasia (BPH) is a progressive condition in aging men that is characterized by the enlargement of the periurethral regions of the prostate gland due to nonmalignant proliferations in both the prostate epithelial and stromal compartments [1]. An estimated 50% of men have histologic evidence of BPH by age 50 years and 75% by age 80 years. BPH is rarely fatal but may cause serious life-threatening complications such as acute urinary retention if left untreated.

Molecular mechanism of BPH initiation remains unclear, but inflammation has been considered as a major etiological factor [1]. We showed previously that decreased androgen signaling in the mouse prostate luminal cells during ageing can result in a mild inflammatory tissue microenvironment and modest increase in epithelial proliferation [2]. The human prostate gland is divided into four different anatomic zones: the peripheral zone (PZ), transition zone (TZ), central zone, and anterior fibromuscular stroma. The PZ and TZ prostate are both developmentally derived from the endodermal layer. Interestingly, BPH almost always occurs in TZ but the molecular mechanism underlying the zonal specific prevalence of the disease remains unclear.

The proliferative index of the epithelial cells in human TZ is lower than that in PZ [3]. This implies a distinct tissue microenvironment in TZ. Change of the signaling in the TZ microenvironment during ageing may contribute to BPH initiation. We sought to investigate the unique tissue microenvironment in TZ using mouse models. The mouse and human prostate are both hormonally regulated glands and consist of the same types of epithelial cells. Although they are anatomically quite different, the mouse proximal prostatic duct shares some features with the human TZ prostate. They are both anatomically closer to the urethra and the epithelial cells in the two regions are both relatively replicative quiescent [3,4]. Using a series of molecular, cellular, and genetic analyses, we demonstrated that the stromal cells in mouse proximal prostate express higher levels of diverse canonical and noncanonical Wnt ligands [5]. Wnt5a, a noncanonical Wnt ligand expressed by the prostate stromal cells, directly suppresses the proliferation of epithelial cells. In addition to producing the ligands, the stromal cells in proximal prostate also possess a higher Wnt/β-Catenin signaling than the stromal cells at the tips of prostate ducts (distal prostate). The stromal Wnt/β-Catenin signaling induces the production of TGFβ ligands, which in turn suppresses the proliferation of epithelial cells indirectly. Together, the direct and indirect Wnt-relating signaling mediated by the stromal cells in the proximal prostate maintain a lower proliferative index of the epithelial cells in this specific anatomic region. We also extended our observation in the human prostate and demonstrated that the stromal cells in human TZ express higher levels of *Wnt5a*, *Axin2*, and *TGFβ2/3*.

Our finding offers a potential molecular mechanism accounting for the distinct proliferative potential of the epithelial cells in TZ and PZ. Since TGFβ has been shown to regulate Wnt5a expression [6], it will be interesting to figure out whether the direct and indirect mechanisms mediated by the stromal cells are intertwined and if yes which mechanism plays a dominant role in restricting epithelial proliferation. Ageing, inflammation, and alteration of androgen signaling are all closely associated with the occurrence of BPH. Interestingly, all these etiological factors have been shown to negatively regulate Wnt signaling [7,8]. Therefore, it is reasonable to hypothesize that during ageing or in the presence of chronic inflammation, the stromal mediated Wnt signaling is attenuated, which contributes to the benign proliferation of the prostate epithelial cells and thereby the initiation of BPH. Future molecular and cellular studies using human BPH specimens will reveal whether the stromal mediated Wnt signaling is indeed altered.
